# Multi-Layer Encryption for Secure 6G MIMO-AFDM-IM ISAC Systems

**DOI:** 10.3390/s26123882

**Published:** 2026-06-18

**Authors:** Ruiqi Cao, Yanqun Tang, Caiqin Li, Sitong Li, Yicong Su, Xinyan Ma, Wei Li, Miao Zhang

**Affiliations:** 1 School of Electronics and Communication Engineering, Sun Yat-sen University, Shenzhen 518107, China; caorq3@mail2.sysu.edu.cn (R.C.); licaiqin0310@163.com (C.L.); list27@mail2.sysu.edu.cn (S.L.); 2 Guangdong Provincial Key Laboratory of Sea-Air-Space Communication, Shenzhen 518107, China; 3 Hangzhou Zhiyuan Research Institute Co., Ltd., Hangzhou 310024, China; vanessamxy@163.com; 4 Shanghai Satellite Network Research Institute Co., Ltd., Shanghai 201210, China; 5 State Key Laboratory of Satellite Network, Shanghai 201210, China; 6 School of Electronic Science and Engineering, National University of Defense Technology, Changsha 410073, China; 7 School of Information Science and Engineering, Chongqing Jiaotong University, Chongqing 400074, China; msczz@foxmail.com

**Keywords:** physical-layer security, integrated sensing and communication, multiple-input multiple-output, affine frequency division multiplexing, index modulation

## Abstract

With the emergence of mobile sixth-generation (6G) integrated sensing and communication (ISAC) scenarios, conventional multicarrier waveforms face challenges in maintaining reliable communication and robust physical-layer security. In this paper, we propose a multi-layer encryption multiple-input multiple-output (MIMO) affine frequency division multiplexing (AFDM) with index modulation (IM) scheme, which exploits the inherent flexibility of the AFDM modulation parameter $c_{2}$ and subcarrier IM to construct a multi-dimensional physical-layer security mechanism. To enable sensing and exploit MIMO spatial diversity, a unified downlink MIMO configuration is adopted, where sensing and communication share the same transmit waveform, receive array, and physical propagation environment. The proposed configuration enables multi-dimensional parameter estimation, including delay, Doppler, and angle. The obtained sensing information further assists beamforming design, channel reconstruction, and signal equalization. Furthermore, the base station and user equipment share synchronized secret keys, and a unified detection framework is developed to balance computational complexity and detection accuracy while remaining compatible with the multi-dimensional encryption structure of the MIMO-AFDM-IM system. Simulation results verify the effectiveness of the proposed scheme in mobile scenarios, demonstrating enhanced multi-dimensional sensing accuracy, improved resistance to eavesdropping, and superior communication reliability and energy efficiency (EE).

## 1. Introduction

Sixth-generation (6G) wireless systems are envisioned to provide ultra-reliable and secure connectivity under mobile and heterogeneous environments, while supporting massive Internet-of-Things (IoT) devices and emerging applications [1,2,3,4]. The evolution toward integrated space–air–ground architectures, incorporating terrestrial networks, low Earth orbit satellites, and low-altitude platforms, further intensifies mobility and channel variability [5]. In such scenarios, conventional separated sensing and communication designs become inefficient in terms of spectrum usage, hardware cost, and real-time environmental awareness. Integrated sensing and communication (ISAC) therefore emerges as a fundamental paradigm for 6G systems, enabling spectrum sharing, hardware reuse, and adaptive environment-aware transmission [6,7,8,9,10,11].

Waveform design plays a pivotal role in the realization of ISAC, as the transmitted signal must jointly ensure communication reliability, sensing accuracy, and robustness in mobile scenarios. Orthogonal frequency division multiplexing (OFDM) has been widely adopted in modern communication systems (e.g., LTE, 5G NR, and WiFi) due to its robustness against multipath fading, high spectral efficiency (SE), and low-complexity FFT-based implementation [6,12,13,14,15]. However, in mobile scenarios, Doppler shifts can destroy the subcarrier orthogonality of OFDM, leading to significant inter-carrier interference (ICI). This Doppler sensitivity degrades communication reliability and limits sensing performance in ISAC systems.

Extending the multicarrier principle of OFDM, affine frequency division multiplexing (AFDM) has recently been proposed as a promising candidate waveform [16,17,18,19,20,21,22]. AFDM offers several advantages, including optimal diversity gain, compatibility with FFT-based OFDM architectures, and robustness against Doppler spread. These properties originate from two chirp parameters, $c_{1}$ and $c_{2}$, which determine the signal representation in the discrete affine Fourier (DAF) domain and govern its interaction with the channel delay–Doppler characteristics [16,17,23]. Notably, when $c_{1}=c_{2}=0$, AFDM reduces to conventional OFDM. With properly designed chirp parameters, the waveform enables effective path separation in the DAF domain, thereby ensuring mobility robustness and full diversity gain. The structural flexibility introduced by $c_{1}$ and $c_{2}$ also provides opportunities for enhancing physical-layer security [20,21,22,24]. By shaping the affine Fourier-domain signal structure, waveform-level protection can be incorporated without compromising subcarrier orthogonality [20,21,24]. Existing AFDM security schemes largely exploit this chirp-parameter flexibility. For instance, dynamic pre-chirp-parameter generation based on time-division duplexing channel reciprocity ensures waveform mismatch for eavesdroppers (Eves) [20]. High-dimensional permutation of chirp-induced basis functions expands the effective parameter search space [21], while symbol-level random hopping forces real-time parameter estimation at the Eve [22]. Despite their effectiveness, these methods share a common design principle: security is primarily achieved through chirp-parameter manipulation. However, since $c_{1}$ and $c_{2}$ simultaneously determine diversity gain, delay–Doppler resolution, and channel robustness, they must satisfy communication and sensing performance constraints [16,18]. This inherent coupling limits the degrees of freedom available for security-oriented optimization. To further enhance physical-layer security, cryptographic techniques and hybrid chaotic and structural encryption frameworks have been proposed in the literature [25,26,27].

To overcome this limitation, additional structural dimensions must be explored. One promising approach is index modulation (IM), which conveys information through the activation patterns of subcarriers [28,29], antennas [30], or other transmission entities [31,32], rather than solely through conventional symbol mapping. By partially activating transmission entities, IM enhances communication reliability and energy efficiency (EE) [28,29,30,31,32]. Meanwhile, the associated discrete index states provide additional signaling degrees of freedom that are largely independent of the fundamental waveform parameters. As a natural extension of the multicarrier framework, AFDM can incorporate IM, where information is embedded into subcarrier activation patterns. This index-domain signaling adds a layer of structural uncertainty, which enhances resistance to unauthorized detection without interfering with the underlying chirp-parameter configuration.

To further enhance sensing and communication performance, multiple-input multiple-output (MIMO) architectures leverage the spatial domain via multiple antennas, facilitating angle estimation and adaptive transmit and receive beamforming [33,34]. For example, a tensor-based angle–delay–Doppler estimation scheme for MIMO-AFDM-ISAC systems was proposed in Ref. [33]. In this work, structured cyclic prefix decomposition is employed to ensure identifiable and accurate parameter recovery under both near-field and far-field conditions. Furthermore, a MIMO-based affine Fourier transform multicarrier waveform integrated localization framework was developed in Ref. [34], where continuous delay and Doppler modeling enables high-precision parameter estimation, approaching the Cramér–Rao lower bound (CRLB).

From the communication perspective, the spatial processing capability of MIMO enhances link reliability and SE through spatial multiplexing and beamforming [18]. Recent studies have addressed key communication challenges in MIMO-AFDM systems. For instance, a diagonally reconstructed pilot-aided channel estimation scheme was proposed in Ref. [18] to suppress inter-Doppler interference in doubly selective channels. Additionally, spatial multiplexing has been combined with IM in Ref. [35] to improve SE while maintaining manageable detection complexity. In MIMO-ISAC systems, sensing and communication are intrinsically coupled: the estimated spatial parameters facilitate adaptive beamforming, while communication signals simultaneously serve as probing waveforms. Effective coordination of spatial-domain processing is therefore critical for mutual performance enhancement.

In light of the above analyses, this paper focuses on a specific problem: how to introduce structural physical-layer protection into a mobile MIMO-AFDM ISAC waveform while still exploiting sensing information for communication recovery [36,37,38,39,40]. The proposed design is not intended as a stand-alone cryptographic protocol; rather, it provides waveform- and index-domain protection that complements upper-layer key agreement. The major contributions of this paper are summarized as follows.

1. Unified Sensing-Assisted MIMO-AFDM-IM Architecture: We develop a single-stream beamformed MIMO-AFDM-IM ISAC scheme, in which sensing and communication share the same AFDM waveform, receive array, and propagation environment. The estimated angle–delay–Doppler parameters are exploited for beamforming, channel reconstruction, and equalization while maintaining the Doppler robustness of AFDM.
2. Decoupled Hybrid-Domain Physical-Layer Protection: We combine key-dependent subcarrier index permutation with subcarrier-wise chirp-parameter permutation. The index domain conceals the active-subcarrier support, whereas the chirp domain distorts the affine Fourier-domain phase structure under an incorrect key. This decouples security-oriented structural uncertainty from the AFDM parameter choices used for delay–Doppler robustness.
3. Cascaded Sensing and Communication Receiver: A cascaded receiver is developed to estimate AOA, delay, AOD, Doppler, and gain from AFDM sensing symbols. The reconstructed parameters are then used to form the beamformed equivalent channel for encrypted AFDM-IM detection.
4. Analytical and Numerical Characterization with Explicit Scope: We analyze key-space expansion, parameter-mismatch effects, CRLB, BER behavior, SE, and computational complexity. The BER derivation is positioned as an analytical benchmark for generalized MIMO-AFDM-IM error behavior, while simulations evaluate the proposed secure single-stream ISAC receiver under ideal and estimated CSI conditions.

The rest of this paper is organized as follows. In Section 2, the ISAC channel model and AFDM signal modeling are first described, followed by the introduction of different encryption techniques. In Section 3, the sensing receiver is discussed, including the cascaded multi-domain parameter estimation framework and methods for angle of arrival (AOA), time-delay, and joint angle of departure (AOD)-Doppler-Gain estimation. Section 4 focuses on the communication receiver, addressing waveform decryption and sensing-assisted channel reconstruction. In Section 5, performance analysis is conducted, covering security analysis, CRLB analysis, BER analysis, SE, and computational complexity. In Section 6, simulation results are presented, including security, sensing, and communication performance evaluations. Finally, Section 7 concludes the paper.

Notations: The notations ${\left(\cdot \right)}^{T},{\left(\cdot \right)}^{H},{\left(\cdot \right)}^{-1}$, and ${\left(\cdot \right)}^{†}$ denote the transpose, conjugate transpose, matrix inversion, and pseudo-inverse operations, respectively. ⌊·⌋ and ∥·∥ denote the floor function and the Euclidean norm, respectively, and ${∥A∥}_{F}$ denotes the Frobenius norm of matrix A. Re(·) denotes the real part of a complex number, vector, or matrix, and ∠(·) denotes the phase angle of a complex number. C(n,m) denotes the combination, i.e., selecting *m* elements from *n*. diag(·) transforms a vector into a diagonal matrix. vec(·) yields a column vector, and mat(·) reshapes a vector into a matrix by collecting rows according to their indices. δ(·) denotes the Dirac delta function. $C^{M\times N}$ denotes the set of M×N complex-valued matrices, and $I_{N}$ denotes the N×N identity matrix. $\mathrm{CN}(0,\sigma^{2})$ denotes a circularly symmetric complex Gaussian distribution with zero mean and variance $\sigma^{2}$. E{·} denotes the expectation operator, and ⊗ denotes the Kronecker product. $j=\sqrt{-1}$ denotes the imaginary unit.

## 2. Preliminaries and System Model

**Figure 1:**
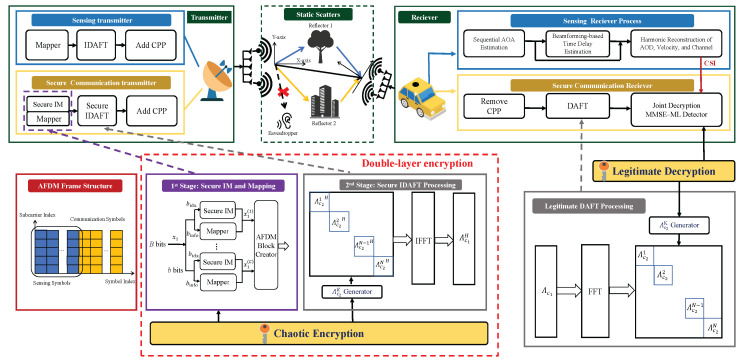
Structure of the proposed secure MIMO-AFDM-ISAC scheme.

The overall architecture of the proposed secure MIMO-AFDM-ISAC framework is illustrated in Figure 1.

As shown in Figure 1, the proposed scheme adopts a unified downlink transceiver architecture, in which sensing and communication share the same transmit waveform, receive array, and physical propagation environment. An AFDM frame comprises $M_{s}=M_{R}+M_{C}$ symbols, where $M_{R}$ and $M_{C}$ represent the numbers of sensing and communication symbols, respectively. Each AFDM symbol occupies *N* subcarriers. For key synchronization, we assume that the BS and UE have completed an authenticated key agreement procedure before data transmission. The shared master key, together with a public frame counter or nonce, is used to locally generate the same index permutation and chirp-parameter vector at both ends, so no additional transmission of the instantaneous permutation is required. Thus, the control overhead is mainly associated with maintaining the common counter or nonce and resynchronizing it when frame loss occurs, rather than distributing full subcarrier-index or chirp-parameter assignments.

The sensing symbols are used by the communication receiver to estimate the delay, Doppler, angular parameters, and channel coefficients of the shared doubly selective channel. These estimated parameters are subsequently utilized for beamforming, channel reconstruction, and communication signal recovery.

To enhance transmission confidentiality, a multi-layer physical-layer encryption mechanism is incorporated into the communication process, leveraging modulation chirp parameters and subcarrier IM.

### 2.1. ISAC Channel Model

The system operates at carrier frequency $f_{C}$ in a downlink MIMO configuration. The base station (BS) employs $N_{t}$ transmit antennas, and the user equipment (UE) employs $N_{r}$ receive antennas, both arranged as uniform linear arrays with half-wavelength spacing.

Sensing and communication share the same physical channel realization and receive array, but they adopt different mathematical representations according to their functional objectives. Sensing focuses on multi-symbol parameter estimation, whereas communication performs data detection over the corresponding doubly selective channel.

#### 2.1.1. Geometric Propagation Model

**Figure 2:**
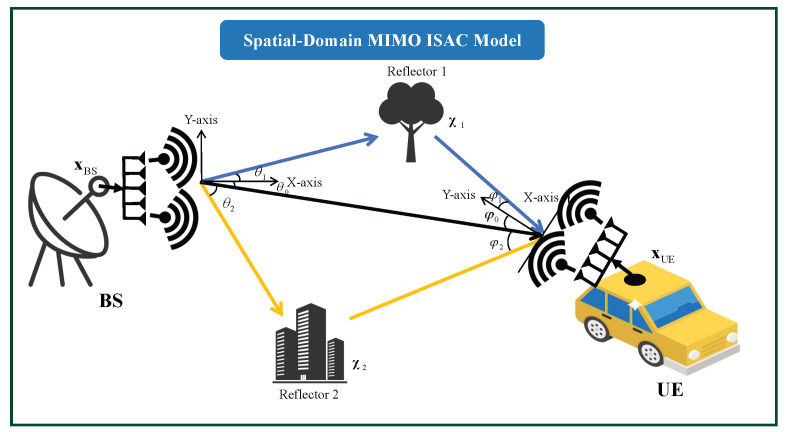
Illustration of MIMO geometric propagation system.

We adopt a two-dimensional geometric propagation model to characterize the spatial structure of the MIMO channel, as shown in Figure 2.

The BS is located at $x_{\mathrm{BS}}\in R^{2}$ and the UE at $x_{\mathrm{UE}}\in R^{2}$, with UE velocity $v_{\mathrm{UE}}\in R^{2}$ and array orientation angle $\phi_{\mathrm{UE}}\in R$. The channel consists of *P* propagation paths. The index p=0 denotes the geometrically direct path between the BS and the UE, while p=1,…,P−1 correspond to reflected or scattered paths. The position of the *p*-th scatterer is denoted by $\chi_{p}\in R^{2}$ for p≥1.

The path delays are given by

(1) $$\begin{array}{cc} \tau_{0} & =\frac{∥x_{\mathrm{UE}}-x_{\mathrm{BS}}{∥}_{2}}{c}, \end{array}$$

(2) $$\begin{array}{cc} \tau_{p} & =\frac{∥x_{\mathrm{UE}}-\chi_{p}{∥}_{2}+{∥x_{\mathrm{BS}}-\chi_{p}∥}_{2}}{c},\;p\geq 1. \end{array}$$

where *c* denotes the speed of light.

The corresponding AoA and AoD parameters are defined as

(3) $$\begin{array}{cc} \vartheta_{0} & =\mathrm{atan2}\;\left(x_{\mathrm{BS},y}-x_{\mathrm{UE},y},x_{\mathrm{BS},x}-x_{\mathrm{UE},x}\right)-\phi_{\mathrm{UE}}, \end{array}$$

(4) $$\begin{array}{cc} \vartheta_{p} & =\mathrm{atan2}\;\left(\chi_{p,y}-x_{\mathrm{UE},y},\chi_{p,x}-x_{\mathrm{UE},x}\right)-\phi_{\mathrm{UE}},\;p\geq 1, \end{array}$$

(5) $$\begin{array}{cc} \varphi_{0} & =\mathrm{atan2}\;\left(x_{\mathrm{UE},y}-x_{\mathrm{BS},y},x_{\mathrm{UE},x}-x_{\mathrm{BS},x}\right), \end{array}$$

(6) $$\begin{array}{cc} \varphi_{p} & =\mathrm{atan2}\;\left(\chi_{p,y}-x_{\mathrm{BS},y},\chi_{p,x}-x_{\mathrm{BS},x}\right),\;p\geq 1, \end{array}$$

where atan2(y,x) denotes the four-quadrant inverse tangent function, which returns the planar direction angle of a two-dimensional vector.

The continuous-time angular Doppler frequency of the *p*-th path is

(7) $$\mu_{p}=2\pi \frac{f_{C}v_{p}}{c}.$$

where the radial velocities are

(8) $$v_{0}=\frac{v_{\mathrm{UE}}^{⊤}\left(x_{\mathrm{BS}}-x_{\mathrm{UE}}\right)}{∥x_{\mathrm{BS}}-x_{\mathrm{UE}}{∥}_{2}}.$$

(9) $$v_{p}=\frac{v_{\mathrm{UE}}^{⊤}\left(\chi_{p}-x_{\mathrm{UE}}\right)}{∥\chi_{p}-x_{\mathrm{UE}}{∥}_{2}},\;p\geq 1.$$

For compact notation, define $\vartheta =\mathrm{vec}(\vartheta_{0},\ldots ,\vartheta_{P-1})$, $\varphi =\mathrm{vec}(\varphi_{0},\ldots ,\varphi_{P-1})$, $\tau =\mathrm{vec}(\tau_{0},\ldots ,\tau_{P-1})$, and $\mu =\mathrm{vec}(\mu_{0},\ldots ,\mu_{P-1})$. The complex channel gain of the *p*-th path is denoted by $h_{p}\in C$ and modeled as

(10) $$h_{p}=\sqrt{\beta_{p}}g_{p},\;g_{p}∼\mathrm{CN}\left(0,1/P\right),$$

where $g_{p}$ represents the small-scale Rayleigh fading coefficient and $\beta_{p}$ denotes the large-scale attenuation coefficient. In the normalized baseline setting, $\beta_{p}=1$ is adopted for all paths to ensure average power normalization. When distance-dependent path loss and shadowing are included, $\beta_{p}$ can be specified as $\beta_{p}=d_{p}^{-\alpha }10^{\xi_{p}/10}$, where $d_{p}$ is the propagation distance, α is the path-loss exponent, and $\xi_{p}∼N\left(0,\sigma_{\mathrm{sh}}^{2}\right)$ models log-normal shadowing in dB. Thus, the gain follows $h_{p}∼\mathrm{CN}\left(0,\beta_{p}/P\right)$ under the adopted statistical fading model.

The adopted channel model is a frame-wise geometric multipath fading model rather than a single-path idealization. The parameter *P* accounts for multiple propagation paths, while the four-dimensional parameter set $\left\{\vartheta_{p},\varphi_{p},\tau_{p},\mu_{p}\right\}$ captures the spatial, delay, and Doppler characteristics of each path in the MIMO configuration. This modeling approach follows the mainstream AFDM and AFDM-ISAC literature, where the performance advantage of AFDM over OFDM is commonly evaluated under doubly selective multipath channels [16,17,18,28,33]. In this paper, the channel is assumed quasi-static within one AFDM frame and updated across Monte Carlo realizations, so the analysis focuses on delay–Doppler–spatial coupling, AFDM waveform robustness, and secure ISAC design.

#### 2.1.2. Sensing MIMO Channel Representation

Based on the geometric model in Section 2.1.1, the sensing channel can be expressed in a structured parametric form. For sensing functionality, the BS transmits $M_{R}$ AFDM sensing symbols, and the UE-side receiver observes the resulting angle–delay–Doppler responses over multiple symbols.

Since AFDM is a linear unitary transform of time-domain samples, the channel can be equivalently represented in the subcarrier–symbol domain. For the *n*-th subcarrier and the *m*-th sensing symbol, the baseband MIMO channel matrix $H_{n,m}\in C^{N_{r}\times N_{t}}$ admits the decomposition

(11) $$H_{n,m}=A_{R}^{H}\left(\vartheta \right)\;{\tilde{H}}_{n,m}\left(\tau ,\mu ,h\right)\;A_{T}\left(\varphi \right),$$

where the diagonal matrix ${\tilde{H}}_{n,m}\in C^{P\times P}$ is given by

(12) $${\tilde{H}}_{n,m}=\mathrm{diag}\left\{h_{p}\exp\;\left(-j\left(\omega_{n}\tau_{p}-mP_{S}\mu_{p}\right)\right)\;|\;p=0,\ldots ,P-1\right\}.$$

Here, $\omega_{n}$ denotes the angular frequency of the *n*-th subcarrier, $P_{S}=NT_{S}$ is the duration of one AFDM sensing symbol, and $\left\{\tau_{p},\mu_{p},h_{p}\right\}$ are defined in the geometric model.

The matrices $A_{R}\left(\vartheta \right)\in C^{P\times N_{r}}$ and $A_{T}\left(\varphi \right)\in C^{P\times N_{t}}$ collect the receive and transmit array steering vectors:

(13) $$\begin{array}{cc} A_{R}\left(\vartheta \right) & =\mathrm{mat}\left[a_{R}^{H}\left(\vartheta_{p}\right)\;|\;p=0,\ldots ,P-1\right], \end{array}$$

(14) $$\begin{array}{cc} A_{T}\left(\varphi \right) & =\mathrm{mat}\left[a_{T}^{H}\left(\varphi_{p}\right)\;|\;p=0,\ldots ,P-1\right], \end{array}$$

where the ULA steering vectors are

(15) $$\begin{array}{cc} a_{R}\left(\vartheta_{p}\right) & =\mathrm{vec}\left[\exp\;\left(-j\pi \left(r-1\right)\sin\vartheta_{p}\right)\;|\;r=1,\ldots ,N_{r}\right], \end{array}$$

(16) $$\begin{array}{cc} a_{T}\left(\varphi_{p}\right) & =\mathrm{vec}\left[\exp\;\left(-j\pi \left(t-1\right)\sin\varphi_{p}\right)\;|\;t=1,\ldots ,N_{t}\right]. \end{array}$$

Equation (11) shows that the sensing channel lies on a structured angle–delay–Doppler manifold induced by the underlying geometry. This parametric structure will be exploited in the subsequent sensing algorithm design.

#### 2.1.3. Communication MIMO Doubly Selective Channel

We now characterize the communication channel corresponding to encrypted data transmission. Although sensing and communication share the same geometric propagation parameters {τ,μ,h,ϑ,φ}, the communication model focuses on the discrete-time input–output relation under doubly selective fading.

Consider an AFDM block with *N* samples per transmit antenna. By stacking all transmit and receive antennas, the overall MIMO channel is represented by

(17) $$H_{\mathrm{MIMO}}\in C^{N_{r}N\times N_{t}N},$$

which admits the block structure

(18) $$H_{\mathrm{MIMO}}=\left[\begin{array}{ccc} H_{1,1} & \ldots & H_{1,N_{t}}\\ \vdots & \ddots & \vdots \\ H_{N_{r},1} & \ldots & H_{N_{r},N_{t}} \end{array}\right],$$

where each element $H_{r,t}$ represents the time-domain channel matrix between the *r*-th RA and the *t*-th TA, with $r=1,\ldots ,N_{r}$. This matrix is expressed as

(19) $$H_{r,t}=\sum_{p=0}^{P-1}h_{p}^{\left[r,t\right]}\Gamma_{{\mathrm{CPP}}_{p}}\Delta_{f_{p}}\Pi^{l_{p}},$$

where *P* represents the number of propagation paths, and $h_{p}^{\left[r,t\right]}$, $l_{p}$, and $f_{p}$ denote the channel gain, delay, and Doppler shift of the *p*-th path, respectively, with p∈{0,…,P−1}.

The channel coefficient $h_{p}^{\left[r,t\right]}$ is determined by the path gain $h_{p}$ and the corresponding receive and transmit array responses. Specifically, it is given by

(20) $$h_{p}^{\left[r,t\right]}=h_{p}\;{\left[a_{R}\left(\vartheta_{p}\right)\right]}_{r}\;{\left[a_{T}\left(\varphi_{p}\right)\right]}_{t}^{*},$$

where ${\left[a_{R}\left(\vartheta_{p}\right)\right]}_{r}$ and ${\left[a_{T}\left(\varphi_{p}\right)\right]}_{t}$ denote the *r*-th and *t*-th entries of the receive and transmit steering vectors, respectively.

For digital signal processing, the continuous-time-delay $\tau_{p}$ and Doppler angular frequency $\mu_{p}$ obtained from the sensing-domain model must be discretized in accordance with the system bandwidth *B* and observation duration $P_{S}$. According to the sampling theorem, the delay resolution is approximately 1/B, while the Doppler resolution is approximately $1/P_{S}$. Accordingly, the continuous parameters are mapped to discrete delay taps while preserving fractional Doppler shifts:

(21) $$l_{p}=\mathrm{round}\left(B\tau_{p}\right).$$

(22) $$f_{p}=\frac{P_{S}\mu_{p}}{2\pi }.$$

where $l_{p}$ denotes the integer delay tap associated with the *p*-th path, and $f_{p}$ represents the normalized (generally fractional) Doppler shift.

The delay tap satisfies $0\leq l_{p}\leq l_{\max}$ with $l_{\max}=\mathrm{round}\left(B\tau_{\max}\right)$, while the normalized Doppler tap lies within $f_{p}\in \left[-f_{\max},f_{\max}\right]$, where $f_{\max}=\mathrm{round}\left(P_{S}\mu_{\max}/2\pi \right)$. Here, $\tau_{\max}$ and $\mu_{\max}$ are determined by the maximum sensing range and maximum relative radial velocity in the geometric model.

The matrix $\Pi \in C^{N\times N}$ is the forward cyclic shift matrix, and

$$\Delta_{f_{p}}≜\mathrm{diag}{\left(e^{-j2\pi f_{p}n}\right)}_{n=0}^{N-1}$$

is the diagonal matrix incorporating the Doppler-induced phase rotations.

Additionally, $\Gamma_{{\mathrm{CPP}}_{p}}$ is defined as

(23) $$\Gamma_{{\mathrm{CPP}}_{p}}=\mathrm{diag}\left(\left\{\begin{array}{cc} e^{-j2\pi c_{1}\left(N^{2}-2N\left(l_{p}-n\right)\right)}, & n<l_{p}\\ 1, & n\geq l_{p} \end{array}\right.\right),$$

where n=0,…,N−1.

Equation (19) indicates that the communication channel is doubly selective, introducing delay dispersion through $\Pi^{\;l_{p}}$ and Doppler dispersion through $\Delta_{f_{p}}$. All parameters are inherited from the unified geometric model, ensuring structural consistency between the sensing and communication representations.

### 2.2. AFDM Signal Model

In the proposed MIMO-AFDM-IM scheme, each transmit antenna adopts AFDM modulation over a block of *N* samples. Let $x\in C^{N\times 1}$ denote the symbol vector in the affine Fourier domain. The corresponding time-domain signal is generated as

(24) $$s=A^{-1}x,\;A=\Lambda_{c_{2}}F_{N}\Lambda_{c_{1}},$$

where $F_{N}$ is the *N*-point unitary DFT matrix, and $\Lambda_{c_{1}}$ and $\Lambda_{c_{2}}$ are diagonal chirp matrices parameterized by real coefficients $c_{1}$ and $c_{2}$, respectively. Since A is unitary, $A^{-1}=A^{H}$.

Thus, AFDM can be interpreted as a chirp-augmented OFDM structure, where quadratic phase rotations are applied before and after the DFT operation. When $c_{1}=c_{2}=0$, the scheme reduces to conventional OFDM.

The discrete-time AFDM waveform is equivalently written as

(25) $$s\left[n\right]=\frac{1}{\sqrt{N}}\sum_{m=0}^{N-1}x\left[m\right]\;e^{j2\pi \left(c_{1}n^{2}+\frac{mn}{N}+c_{2}m^{2}\right)},\;n=0,\ldots ,N-1.$$

To preserve circular convolution under doubly selective channels, a chirp cyclic prefix (CPP) is appended before transmission.

For practical parameter selection, $c_{1}$ is mainly determined by the maximum normalized Doppler spread and the delay–Doppler separability requirement. Following the AFDM full-diversity condition, $c_{1}$ should be large enough to separate paths with different delay–Doppler shifts in the affine Fourier domain; in the simulations, it is set as $c_{1}=\left(2f_{\max}+1\right)/\left(2N\right)$. The parameter $c_{2}$ does not change the diversity order but controls the quadratic phase rotation across subcarriers. In conventional AFDM, $c_{2}$ is usually selected as an irrational number or a rational value much smaller than 1/(2N) to avoid harmful ambiguity. In the proposed secure design, $c_{2}$ is further extended to a key-controlled subcarrier-wise vector generated from a bounded codebook. Therefore, the selection of $c_{2,\max}$ follows two practical criteria: it should be small enough to preserve reliable demodulation for the legitimate receiver with the correct key, and sufficiently large to create strong phase mismatch at the eavesdropper when the key is unknown.

### 2.3. First-Layer Encryption: Subcarrier Index Modulation

The first security layer is implemented at the subcarrier activation level through encrypted IM. Different from conventional AFDM-IM, where the mapping between index bits and active-subcarrier patterns is fixed and publicly known, the proposed scheme introduces a key-dependent permutation into the index selection process, thereby concealing the structural sparsity pattern of transmitted symbols.

Consider the *t*-th transmit antenna. Within one AFDM block of length *N*, the input bit stream of *B* bits is divided into *G* sub-blocks, each occupying $n_{\mathrm{sub}}=N/G$ subcarriers. For the *g*-th sub-block, the corresponding b=B/G bits are partitioned into $b_{\mathrm{idx}}$ index bits and $b_{\mathrm{info}}$ modulation bits. Among the $n_{\mathrm{sub}}$ available subcarriers, $k_{\mathrm{sub}}$ positions are activated according to the index bits.

**Figure 3:**
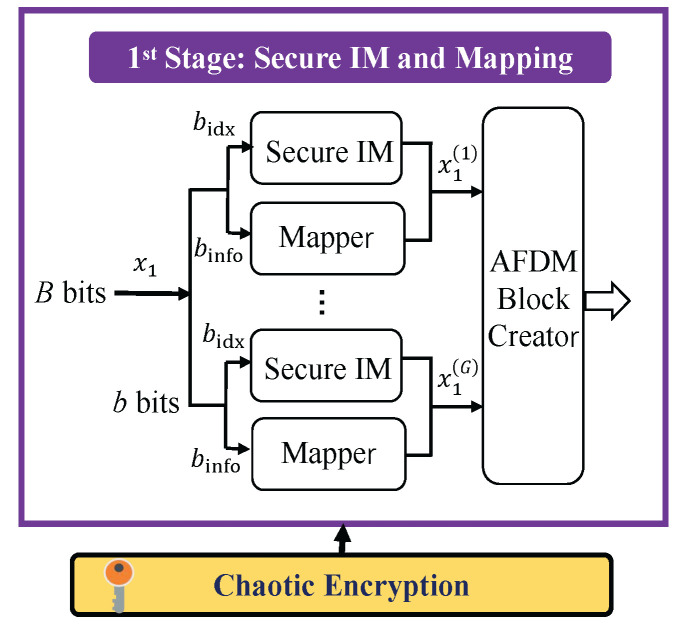
Subcarrier index modulation mechanism.

Let $I_{t}^{g}=\left\{i_{t,1}^{g},\ldots ,i_{t,k_{\mathrm{sub}}}^{g}\right\}\subset \left\{1,\ldots ,n_{\mathrm{sub}}\right\}$ denote the active index set. The $b_{\mathrm{info}}$ bits are mapped to constellation symbols ${\bar{x}}_{t}^{g}={\left[x_{t,1}^{g},\ldots ,x_{t,k_{\mathrm{sub}}}^{g}\right]}^{T}$, which are inserted into the corresponding subcarrier positions to form $x_{t}^{g}\in C^{n_{\mathrm{sub}}\times 1}$ with zero entries elsewhere. After processing all *G* sub-blocks, the complete AFDM-domain vector is constructed as $x_{t}={\left[{\left(x_{t}^{1}\right)}^{T},\ldots ,{\left(x_{t}^{G}\right)}^{T}\right]}^{T}\in C^{N\times 1}$. The principle of the encrypted subcarrier IM mechanism is illustrated in Figure 3.

In conventional IM, the total number of feasible activation patterns is Q=nsubksub. Since only an integer number of index bits can be conveyed, the practical mapper uses $U=2^{\left\lfloor \log_{2}Q\right\rfloor }$ activation patterns for bit-to-index mapping. In the proposed scheme, this ordered set of *U* employed patterns is permuted by a secret-key-controlled bijection $\pi_{K}:\left\{1,\ldots ,U\right\}\to \left\{1,\ldots ,U\right\}$, and the index bits are mapped according to the permuted ordering. In implementation, the employed activation patterns can be stored in a lookup table and accessed through a pseudo-random seed or a key-controlled permutation, so the transmitter does not need to perform an online combinatorial search over all nsubksub patterns. Nevertheless, IM inevitably introduces a complexity–performance trade-off because support detection and index demapping require additional memory access and decision operations. Low-complexity AFDM-IM detection has been studied in related work [28,35]; in the present secure ISAC design, MMSE equalization followed by sub-block-wise ML detection is adopted to provide a reliable performance benchmark. For illustration, consider $n_{\mathrm{sub}}=4$, $k_{\mathrm{sub}}=2$, for which Q=42=6 and thus $b_{\mathrm{idx}}=\left\lfloor \log_{2}6\right\rfloor =2$. Hence, $U=2^{b_{\mathrm{idx}}}=4$ activation patterns are selected for practical index mapping. Assume the permutation $\pi_{K}:\left(1,2,3,4\right)\to \left(3,1,4,2\right)$. To clarify the impact of the key-dependent permutation, a comparison between the conventional and encrypted index mappings is presented in Table 1.

It is observed that identical index bits correspond to different activation patterns once the permutation $\pi_{K}$ is applied. The number of active subcarriers remains unchanged, thus preserving SE, while the structural sparsity pattern becomes unrecoverable without the secret key. For frame-wise operation, $\pi_{K}$ may be refreshed by combining the shared master key with a frame counter or nonce, so the activation-pattern ordering changes across frames without sending the permutation itself.

This encrypted index mapping constitutes the first protection layer of the proposed secure MIMO-AFDM-IM scheme.

### 2.4. Second-Layer Encryption: Modulation and Demodulation Processing

**Figure 4:**
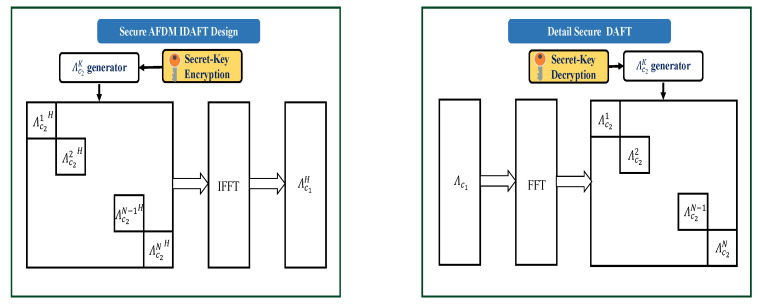
Encrypted modulation and decryption–demodulation structures.

The overall structure of the encrypted modulation and the corresponding decryption–demodulation processing is illustrated in Figure 4.

This layer is implemented at the waveform level by introducing a key-dependent transformation on the AFDM chirp parameter $c_{2}$. In the AFDM signal model, $c_{1}$ ensures delay–Doppler diversity, while $c_{2}$ only induces a quadratic phase rotation across subcarriers without affecting orthogonality or diversity order. This asymmetry allows $c_{2}$ to be exploited for secure waveform design without degrading communication performance.

Specifically, the predefined interval $\left[-c_{2,\max},\;c_{2,\max}\right]$ is uniformly discretized into *N* candidates, forming the public codebook

(26) $$C_{2}=\left\{-c_{2,\max},-c_{2,\max}+\Delta ,\ldots ,c_{2,\max}\right\},\;\Delta =\frac{2c_{2,\max}}{N-1}.$$

Instead of a fixed scalar $c_{2}$, a subcarrier-dependent vector $c_{2}={\left[c_{2}\left[0\right],\ldots ,c_{2}\left[N-1\right]\right]}^{T}$ is generated as a secret-key-controlled permutation of the ordered codebook $C_{2}$. Hence, each codebook element appears exactly once in one AFDM block, while its subcarrier assignment is determined by the secret key. This pseudo-random assignment ensures that, while the parameter range is public, the subcarrier-wise configuration remains unpredictable without the key. The same synchronized seed can also be used to update the chirp-parameter assignment on a frame-wise basis, which maintains legitimate decryption consistency while reducing the risk of stationary waveform-level keys.

The resulting encrypted chirp matrix is

(27) $$\Lambda_{c_{2}}^{K}≜\mathrm{diag}\;{\left(e^{-j2\pi c_{2}\left[m\right]m^{2}}\right)}_{m=0}^{N-1},$$

where the superscript *K* indicates key-dependence, and it replaces the conventional $\Lambda_{c_{2}}$ in AFDM modulation. The transmitted waveform thus becomes

(28) $$s_{K}\left[n\right]=\frac{1}{\sqrt{N}}\sum_{m=0}^{N-1}x\left[m\right]\;e^{j2\pi \left(c_{1}n^{2}+c_{2}\left[m\right]m^{2}+\frac{mn}{N}\right)}.$$

Since the overall transform remains linear and unitary, a legitimate receiver can recover the affine-domain symbols by applying the same key-dependent vector. Without the correct permutation, quadratic phase mismatch disrupts the affine-domain structure, leading to severe symbol distortion.

This waveform-level encryption, together with the previously introduced index-level protection, forms a two-layer physical-layer security mechanism combining structural sparsity concealment and chirp-parameter obfuscation.

## 3. Sensing Receiver

### 3.1. Hierarchical Cascaded Multi-Parameter Joint Estimation Framework

In the proposed MIMO-AFDM-IM ISAC scheme, the communication receiver performs sensing-assisted processing by extracting multipath channel parameters from the received AFDM sensing symbols and reconstructing the angle–delay–Doppler domain channel state. The reconstructed channel is subsequently utilized for beamforming, equalization, and data detection.

Due to the strong coupling among spatial, delay, Doppler, and complex gain parameters, direct high-dimensional joint estimation leads to excessive computational complexity. To address this issue, a cascaded multi-stage estimation framework is adopted. The estimation is carried out progressively across the spatial, frequency, and space–time domains, where AOA, delay, and the remaining parameters are identified in a cascaded manner.

During sensing, the BS transmits $M_{R}$ AFDM sensing symbols.

Let $z_{n,m}\in C^{N_{r}}$ denote the received observation on the *n*-th subcarrier of the *m*-th sensing symbol. Stacking all subcarriers yields the sensing snapshot matrix

(29) $$Z_{m}=\mathrm{mat}{\left[z_{n,m}^{⊤}|n=0,\ldots ,N-1\right]}^{⊤}\in C^{N_{r}\times N}.$$

According to the structured angle–delay–Doppler channel representation in (11) and (12), the sensing snapshot admits the parametric form

(30) $$Z_{m}=\sum_{p=0}^{P-1}h_{p}a_{R}\left(\vartheta_{p}\right)\exp\left(jmP_{S}\mu_{p}\right)a_{T}^{H}\left(\varphi_{p}\right)W_{m}D\left(\tau_{p}\right)+\Omega_{m},$$

where $W_{m}$ collects the transmitted AFDM sensing symbols across subcarriers and $\Omega_{m}$ denotes additive Gaussian noise. The AWGN model provides a baseline setting for evaluating the proposed waveform and encryption mechanism. More non-Gaussian disturbance models, such as Bernoulli–Gaussian impulsive noise, can be incorporated by replacing $\Omega_{m}$ with a mixture-noise term and by using robust estimation or detection methods, although such disturbance-specific receiver designs are beyond the baseline model considered here.

The collection ${\left\{Z_{m}\right\}}_{m=1}^{M_{R}}$ therefore forms a sequence of structured space–frequency snapshots lying on an angle–delay–Doppler manifold, which serves as the input to the subsequent cascaded parameter estimation. The same formulation can accommodate multiple propagation paths or multiple resolvable objects through the summation over p=0,…,P−1. In the cascaded receiver, the MUSIC stage first estimates multiple AOA components, and the delay, AOD, Doppler, and gain are subsequently estimated for each detected path index. The practical multi-object resolvability is limited by the angular aperture, bandwidth, observation duration, and SNR; when two objects have very close angle, range, or Doppler signatures, the corresponding FIM becomes ill-conditioned and the estimation accuracy degrades.

Since the receiver adopts a cascaded rather than fully joint maximum-likelihood estimator, estimation errors may propagate between stages. In particular, an AOA error changes the spatial beamformer used for delay estimation, and the resulting delay bias may further affect the subsequent AOD-Doppler low-rank reconstruction. To reduce this sensitivity, the AOA stage exploits covariance information accumulated over all subcarriers and sensing symbols, while the following stages are performed separately for each detected path to limit cross-path interference. The final reconstructed parameter set can also be used as the initialization for a local residual refinement over a small angle–delay–Doppler neighborhood when higher processing latency is affordable. Therefore, the cascaded design should be interpreted as a complexity–delay trade-off: it avoids prohibitive high-dimensional search, but its accuracy can degrade in low-SNR or strongly coupled multi-object cases.

### 3.2. Angle-of-Arrival Estimation Based on MUSIC

The first stage estimates the AOA parameters by exploiting the orthogonality between signal and noise subspaces.

The spatial correlation matrix is constructed as

(31) $$R=\sum_{m=1}^{M_{R}}Z_{m}Z_{m}^{H}.$$

Applying eigenvalue decomposition, R is partitioned into signal and noise subspaces according to eigenvalue magnitudes, denoted by $U_{S}$ and $U_{\mathrm{noise}}$, respectively.

A threshold-based criterion is employed to determine the number of resolvable paths $P^{♯}$. Let

(32) $$\lambda_{\mathrm{th}}=\varsigma_{\mathrm{th}}\sigma_{\mathrm{noise}}^{2},$$

where $\sigma_{\mathrm{noise}}^{2}$ denotes the noise variance and $\varsigma_{\mathrm{th}}\geq 1$ is a design constant. Eigenvalues satisfying $\lambda_{\ell }\geq \lambda_{\mathrm{th}}$ are classified as signal components, and the number of such eigenvalues determines $P^{♯}$.

For complex-valued array snapshots, the MUSIC pseudo-spectrum is written as

(33) $$P_{\mathrm{MUSIC}}\left(\vartheta \right)=\frac{1}{a_{R}^{⊤}\left(\vartheta \right)U_{\mathrm{noise}}U_{\mathrm{noise}}^{H}a_{R}^{*}\left(\vartheta \right)}.$$

The $P^{♯}$ largest local maxima of $P_{\mathrm{MUSIC}}\left(\vartheta \right)$ are selected as the AOA estimates $\left\{{\hat{\vartheta }}_{0},\ldots ,{\hat{\vartheta }}_{P^{♯}-1}\right\}$.

The estimated AOAs are subsequently utilized to construct angle-selective beamformers, facilitating structured delay estimation in the next stage.

### 3.3. Time-Delay Estimation Based on Spatial Selection

Given the estimated AOAs $\left\{{\hat{\vartheta }}_{0},\ldots ,{\hat{\vartheta }}_{P^{♯}-1}\right\}$, the delay parameters are detected sequentially.

For the *p*-th path, an angle-selective beamformer $s_{p}\in C^{N_{r}}$ is constructed such that

(34) $$s_{p}^{H}a_{R}\left({\hat{\vartheta }}_{p}\right)=1,$$

which enforces unit response toward the desired direction while suppressing other spatial components.

Applying the beamformer to the received sensing symbols $\left\{Z_{m}\right\}$, the multi-parameter coupling among gain, AOD, and Doppler is absorbed into nuisance variables. By exploiting the linear structure of the AFDM sensing model, these nuisance parameters can be eliminated in closed form, leading to a delay-dependent quadratic form.

After elimination, the delay estimation reduces to a one-dimensional spectral search

(35) $${\hat{\tau }}_{p}=\arg\max_{\tau_{p}}a_{R}^{⊤}\left({\hat{\vartheta }}_{p}\right)Q^{-1}\left(\tau_{p}\right)a_{R}^{*}\left({\hat{\vartheta }}_{p}\right),$$

where

(36) $$Q\left(\tau_{p}\right)=\sum_{m=1}^{M_{R}}Z_{m}^{*}\left(I-D\left(\tau_{p}\right)W_{m}^{⊤}{\left(W_{m}^{*}W_{m}^{⊤}\right)}^{-1}W_{m}^{*}D^{H}\left(\tau_{p}\right)\right)Z_{m}^{⊤}.$$

The optimal ${\hat{\tau }}_{p}$ is obtained via a one-dimensional spectrum search. Repeating the above procedure for $p=0,\ldots ,P^{♯}-1$ yields the delay estimates of all detected paths.

### 3.4. Joint Estimation of Angle-of-Departure and Doppler Based on Low-Rank Decomposition

Given the angle–delay estimates ${\left\{\left({\hat{\vartheta }}_{p},{\hat{\tau }}_{p}\right)\right\}}_{p=0}^{P^{♯}-1}$, the remaining parameters, namely the AOD $\varphi_{p}$, Doppler $\mu_{p}$, and complex gain $h_{p}$, are reconstructed by exploiting the space–time harmonic structure of AFDM sensing signals.

The nuisance parameter vectors associated with the delay-domain processing are expressed as

(37) $${\hat{\beta }}_{p,m}={\left(W_{m}^{*}W_{m}^{⊤}\right)}^{-1}W_{m}^{*}D^{H}\left({\hat{\tau }}_{p}\right)Z_{m}^{⊤}{\hat{s}}_{p}^{*},\;m=1,\ldots ,M_{R},$$

where ${\hat{s}}_{p}$ denotes the angle-selective beamformer constructed in the delay estimation stage.

Stacking $\left\{{\hat{\beta }}_{p,m}\right\}$ forms the equivalent matrix

(38) $${\hat{\Psi }}_{p}=\mathrm{mat}{\left[{\hat{\beta }}_{p,m}^{⊤}|m=1,\ldots ,M_{R}\right]}^{⊤}\in C^{N_{t}\times M_{R}}.$$

According to the AFDM signal model, ${\hat{\Psi }}_{p}$ admits a rank-one structure

(39) $${\hat{\Psi }}_{p}=h_{p}a_{T}^{*}\left(\varphi_{p}\right)u_{p}^{⊤}\left(\mu_{p}\right),$$

with

(40) $$u_{p}\left(\mu_{p}\right)=\mathrm{vec}\left[\exp\;\left(jmP_{S}\mu_{p}\right)|m=1,\ldots ,M_{R}\right]\in C^{M_{R}}.$$

Since ${\hat{\Psi }}_{p}$ is separable in the spatial and temporal dimensions, the AOD and Doppler can be estimated via the rotational invariance of its dominant rank-one component. Let ${\hat{u}}_{p}\in C^{N_{t}}$ and ${\hat{v}}_{p}\in C^{M_{R}}$ denote the principal left and right singular vectors of ${\hat{\Psi }}_{p}$, respectively. Define $I_{\mathrm{ROW}}^{▵}$ and $I_{\mathrm{ROW}}^{\nabla }$ as row-selection operators extracting two overlapped subarrays along the spatial dimension, and $I_{\mathrm{COL}}^{▵}$ and $I_{\mathrm{COL}}^{\nabla }$ as column-selection operators extracting two overlapped segments along the temporal dimension. Then the spatial and temporal phase rotations satisfy

(41) $$\exp\;\left(j\pi \sin\varphi_{p}\right)={\left(I_{\mathrm{ROW}}^{▵}{\hat{u}}_{p}\right)}^{†}\left(I_{\mathrm{ROW}}^{\nabla }{\hat{u}}_{p}\right),$$

(42) $$\exp\;\left(jP_{S}\mu_{p}\right)={\left(I_{\mathrm{COL}}^{▵}{\hat{v}}_{p}\right)}^{†}\left(I_{\mathrm{COL}}^{\nabla }{\hat{v}}_{p}\right).$$

Accordingly, the estimates are obtained as

(43) $${\hat{\varphi }}_{p}=\arcsin\left(\frac{∠\;\left({\left(I_{\mathrm{ROW}}^{▵}{\hat{u}}_{p}\right)}^{†}\left(I_{\mathrm{ROW}}^{\nabla }{\hat{u}}_{p}\right)\right)}{\pi }\right),$$

(44) $${\hat{\mu }}_{p}=\frac{1}{P_{S}}∠\;\left({\left(I_{\mathrm{COL}}^{▵}{\hat{v}}_{p}\right)}^{†}\left(I_{\mathrm{COL}}^{\nabla }{\hat{v}}_{p}\right)\right).$$

### 3.5. Complex Gain Estimation Based on Least-Squares Projection

Finally, the complex path gain is obtained via least-squares projection

(45) $${\hat{h}}_{p}=\frac{a_{T}^{⊤}\left({\hat{\varphi }}_{p}\right){\hat{\Psi }}_{p}u_{p}^{*}\left({\hat{\mu }}_{p}\right)}{∥a_{T}\left({\hat{\varphi }}_{p}\right){∥}_{2}^{2}{∥u_{p}\left({\hat{\mu }}_{p}\right)∥}_{2}^{2}}.$$

Repeating the above procedure for all detected paths yields the reconstructed multipath channel parameters. After the above cascaded processing, the multipath parameter set ${\left\{{\hat{\vartheta }}_{p},{\hat{\tau }}_{p},{\hat{\varphi }}_{p},{\hat{\mu }}_{p},{\hat{h}}_{p}\right\}}_{p=0}^{P^{♯}-1}$ is obtained.

The reconstructed channel matrix is subsequently incorporated into the AFDM-IM communication receiver for sensing-assisted equalization and symbol detection, thereby improving robustness under multipath fading conditions.

## 4. Communication Receiver

### 4.1. Waveform Decryption and Beamformed Equivalent Channel

The spatial-domain channel coefficient between the *t*-th transmit antenna and the *r*-th receive antenna for the *p*-th path is expressed as

(46) $$h_{p}^{\left[r,t\right]}=h_{p}\;{\left[a_{R}\left(\vartheta_{p}\right)\right]}_{r}\;{\left[a_{T}\left(\varphi_{p}\right)\right]}_{t}^{*},$$

where ${\left[a_{R}\left(\vartheta_{p}\right)\right]}_{r}$ and ${\left[a_{T}\left(\varphi_{p}\right)\right]}_{t}$ denote the corresponding entries of the receive and transmit steering vectors, respectively.

The proposed practical implementation adopts single-stream transmission with transmit and receive beamforming vectors $w_{t}$ and $w_{r}$. All transmit antennas emit the same AFDM-IM data stream after transmit beamforming, and the receive array performs beam combining before equalization and detection.

After beamforming, the effective gain of the *p*-th propagation path becomes

(47) $${\tilde{h}}_{p}=w_{r}^{H}a_{R}\left(\vartheta_{p}\right)\;h_{p}\;a_{T}^{H}\left(\varphi_{p}\right)w_{t}.$$

which compresses the spatial MIMO channel into an equivalent single-input single-output linear time-varying channel.

The corresponding SISO time-varying channel kernel is therefore

(48) $${\tilde{H}}_{\mathrm{SISO}}=\sum_{p=0}^{P-1}{\tilde{h}}_{p}\Gamma_{{\mathrm{CPP}}_{p}}\Delta_{f_{p}}\Pi^{l_{p}}.$$

Applying waveform decryption and DAFT processing yields the affine-domain equivalent SISO channel

(49) $$H_{\mathrm{eff},\mathrm{SISO}}=\Lambda_{c_{2,K}}F\Lambda_{c_{1}}{\tilde{H}}_{\mathrm{SISO}}\Lambda_{c_{1}}^{H}F^{H}\Lambda_{c_{2,K}}^{H}.$$

The received affine-domain signal is therefore

(50) $$y=H_{\mathrm{eff},\mathrm{SISO}}x+w,$$

where $x\in C^{N\times 1}$ denotes the transmitted AFDM-IM block.

### 4.2. Sensing-Assisted Channel Reconstruction and Detection

After completing the cascaded sensing stages, the estimated multipath parameter set

$${\left\{{\hat{\vartheta }}_{p},{\hat{\tau }}_{p},{\hat{\varphi }}_{p},{\hat{\mu }}_{p},{\hat{h}}_{p}\right\}}_{p=0}^{P^{♯}-1}$$

is obtained.

For digital implementation, the continuous delay and Doppler parameters are discretized as

(51) $${\hat{l}}_{p}=\mathrm{round}\left(B{\hat{\tau }}_{p}\right).$$

(52) $${\hat{f}}_{p}=\frac{P_{S}{\hat{\mu }}_{p}}{2\pi }.$$

Based on the estimated parameters, the reconstructed SISO channel kernel is

(53) $${\hat{\tilde{H}}}_{\mathrm{SISO}}=\sum_{p=0}^{P^{♯}-1}{\hat{\tilde{h}}}_{p}\Gamma_{{\mathrm{CPP}}_{p}}\Delta_{{\hat{f}}_{p}}\Pi^{{\hat{l}}_{p}},$$

where

(54) $${\hat{\tilde{h}}}_{p}=w_{r}^{H}a_{R}\left({\hat{\vartheta }}_{p}\right){\hat{h}}_{p}a_{T}^{H}\left({\hat{\varphi }}_{p}\right)w_{t}.$$

The affine-domain estimated effective channel is therefore

(55) $${\hat{H}}_{\mathrm{eff},\mathrm{SISO}}=\Lambda_{c_{2,K}}F\Lambda_{c_{1}}{\hat{\tilde{H}}}_{\mathrm{SISO}}\Lambda_{c_{1}}^{H}F^{H}\Lambda_{c_{2,K}}^{H}.$$

Let the channel reconstruction error be denoted by $\Delta H_{\mathrm{eff}}={\hat{H}}_{\mathrm{eff},\mathrm{SISO}}-H_{\mathrm{eff},\mathrm{SISO}}$. Such an error may originate from residual AOA, AOD, delay, Doppler, or gain estimation errors, and it directly produces beamforming mismatch and residual interference after equalization. The waveform decryption itself is still determined by the synchronized secret index and chirp assignments, but the reliability of the recovered bit stream depends on the accuracy of the reconstructed channel used by the equalizer. Residual synchronization error, phase noise, or hardware impairments can be interpreted in the same receiver chain as additional channel–model mismatch, and their detailed impairment-specific modeling is beyond the present scope.

An MMSE equalizer based on the estimated channel is adopted:

(56) $$\hat{x}={\hat{H}}_{\mathrm{eff},\mathrm{SISO}}^{H}{\left({\hat{H}}_{\mathrm{eff},\mathrm{SISO}}{\hat{H}}_{\mathrm{eff},\mathrm{SISO}}^{H}+\frac{N_{0}}{E_{b}}I_{N}\right)}^{-1}y.$$

Subsequently, sub-block-wise ML detection is performed, and the detected symbols are demapped to recover the transmitted bit stream.

## 5. Performance Analysis

### 5.1. Security Analysis

In this subsection, we analyze the security performance of the proposed dual-layer encrypted single-stream MIMO-AFDM-IM system. The encryption consists of two structurally independent mechanisms, namely subcarrier index modulation and modulation-domain chirp-parameter encryption. Here, the term “encryption” refers to physical-layer structural obfuscation implemented through key-controlled waveform and index mappings, rather than a proof of semantic security in the cryptographic sense. Accordingly, the following analysis focuses on key-space expansion, parameter mismatch, random-guessing probability, and detection-level BER degradation at the Eve. Formal information-theoretic secrecy metrics, such as secrecy capacity, equivocation, or mutual-information leakage, require a separate adversarial channel model and are beyond the present waveform-level security analysis.

#### 5.1.1. Discussion on Resistance to Known-Plaintext and Chosen-Plaintext Attacks

Beyond passive eavesdropping and brute-force parameter search, stronger attack models such as known-plaintext attacks (KPAs) and chosen-plaintext attacks (CPAs) should also be considered. Following Kerckhoffs’s principle, we assume that the eavesdropper knows all public system parameters, including the AFDM block size, modulation format, IM configuration, and the admissible chirp-parameter set, but does not know the secret index permutation or the subcarrier-wise chirp assignment. In this adversarial model, Eve may observe or inject a limited number of plaintext–ciphertext pairs, but does not control the authenticated key agreement procedure or the frame counter or nonce used to refresh the hidden mappings.

In the first encryption layer, the mapping between the index bits and the active-subcarrier patterns is randomized by a secret permutation. Therefore, even when part of the plaintext structure is known, the eavesdropper still has to infer the concealed correspondence between the observed sparse support and the original index mapping. In the second encryption layer, the subcarrier-wise chirp-parameter vector further randomizes the affine Fourier-domain phase structure. A mismatch in the chirp assignment introduces quadratic phase distortion over the AFDM block, which propagates through the demodulation process and significantly degrades unauthorized detection.

Nevertheless, under long-term reuse of the same hidden mappings, accumulated plaintext–ciphertext pairs may still provide partial constraints on the underlying encryption structure. Accordingly, practical implementations should avoid indefinite reuse of an identical hidden mapping and should adopt periodic or frame-wise refresh when long observation windows are expected. Therefore, the current scheme is more accurately characterized as providing physical-layer resistance against passive eavesdropping, parameter mismatch, and brute-force search, while increasing the difficulty of KPA and CPA compared with single-layer or fixed-structure schemes. Its resistance to KPA and CPA can be further strengthened by introducing frame-varying secret updates, such as regenerating the index permutation and chirp-parameter vector from a master key together with a per-frame random seed, nonce, or sensing-assisted dynamic parameter. In this way, identical plaintexts no longer produce stationary encrypted structures across different AFDM blocks, preventing the eavesdropper from exploiting repeated observations for cryptanalysis.

Based on the above security model, the single-layer and dual-layer security characteristics of the proposed design are analyzed as follows.

#### 5.1.2. Single-Layer Security Characteristics

Subcarrier index modulation (corresponding to Section 2.3): For each sub-block, the total number of feasible activation patterns is Q=nsubksub. Since only an integer number of index bits can be conveyed, the practical IM mapper uses $U=2^{\left\lfloor \log_{2}Q\right\rfloor }$ activation patterns for bit-to-index mapping. A secret permutation over these *U* employed patterns yields U! possible mappings. If independent permutations are used across the *G* sub-blocks, the corresponding key space becomes $K_{\mathrm{idx}}={\left(U!\right)}^{G}$. Due to factorial growth, exhaustive search becomes computationally infeasible for practical system dimensions (e.g., even for moderate U=8 and G=4, the key space already exceeds $10^{20}$), while the random-guess success probability ${\left(1/U!\right)}^{G}$ rapidly approaches zero. If the permutation is incorrect, support detection in the affine-frequency domain fails, and under optimal detection with uniform priors, the index bits become statistically independent of the transmitted bits, yielding ${\mathrm{BER}}_{\mathrm{index}}\to 0.5$. Since the AFDM phase structure remains intact, modulation symbols may still retain partial correlation with the transmitted constellation, as the quadratic phase structure is not distorted. Hence, index-only encryption mainly randomizes index bits, while modulation bits may exhibit limited residual detectability.

Meanwhile, IM inherently enhances structural uncertainty of the waveform. Besides security improvement, it increases SE via activation-based bit embedding, as analytically quantified in Section 5.4, improves EE due to sparse transmission, and can enhance reliability by reducing inter-subcarrier interference. The permutation mechanism strengthens this structural diversity without degrading these performance gains.

Modulation-domain chirp parameter encryption (corresponding to Section 2.4): In the second layer, the scalar parameter $c_{2}$ is extended to a subcarrier-dependent vector $c_{2}={\left[c_{2}\left[0\right],\ldots ,c_{2}\left[N-1\right]\right]}^{T}$ generated by a key-controlled permutation of a discretized codebook. Since the operation corresponds to a full permutation over *N* parameters, the effective key space equals $K_{\mathrm{phase}}=N!$, which grows factorially with the number of subcarriers. For a random permutation, the expected number of correctly matched positions (fixed points) is one. Thus, for large *N*, almost all subcarrier parameters are mismatched under an incorrect key [32].

AFDM modulation contains quadratic phase terms of the form $e^{j2\pi c_{2}\left[m\right]m^{2}}$. With an incorrect permutation, the residual phase becomes $e^{j2\pi \left(c_{2}\left[m\right]-{\tilde{c}}_{2}\left[m\right]\right)m^{2}}$, whose magnitude scales with $m^{2}$. Given that nearly all positions are mismatched, high-index subcarriers experience significantly amplified phase distortion.

Unlike IM, this distortion is global rather than localized. Quadratic phase mismatch propagates through the transform-domain detection process and affects the entire symbol block, driving the overall BER toward 0.5 under optimal detection. Successful decoding therefore requires essentially complete recovery of the quadratic phase permutation.

To further quantify the security of the second-layer chirp-parameter encryption, consider an Eve that randomly guesses the subcarrier-wise permutation of the true chirp-parameter vector without access to the secret key. Let the true chirp-parameter sequence be

(57) $$c_{2}={\left[c_{2}\left[0\right],c_{2}\left[1\right],\ldots ,c_{2}\left[N-1\right]\right]}^{T},$$

and let the guessed sequence be

(58) $${\tilde{c}}_{2}={\left[{\tilde{c}}_{2}\left[0\right],{\tilde{c}}_{2}\left[1\right],\ldots ,{\tilde{c}}_{2}\left[N-1\right]\right]}^{T}.$$

Define *ℓ* as the number of correctly matched positions between ${\tilde{c}}_{2}$ and $c_{2}$, i.e.,

(59) $$\ell =\sum_{m=0}^{N-1}1\;\left({\tilde{c}}_{2}\left[m\right]=c_{2}\left[m\right]\right),$$

where 1(·) denotes the indicator function. Since Eve’s guess can be regarded as an equiprobable selection from all N! permutations, the probability that exactly *ℓ* positions are guessed correctly is

(60) Pℓ(phase)=NℓDN−ℓN!,

where $D_{N-\ell }$ denotes the number of derangements of N−ℓ elements. Equation (60) shows that the probability of fully recovering the chirp-parameter permutation is

(61) $$P_{N}^{\left(\mathrm{phase}\right)}=\frac{1}{N!},$$

which rapidly approaches zero as *N* increases.

Furthermore, by using the derangement expansion, the probability for N=64 can be written as

(62) $$P_{\ell }^{\left(\mathrm{phase}\right)}=\frac{1}{\ell !}\sum_{k=0}^{64-\ell }\frac{{\left(-1\right)}^{k}}{k!}.$$

The numerical results indicate that the matching probability decreases sharply with *ℓ*. In particular, when ℓ=0 and ℓ=1, the probabilities are both approximately 0.3679; when ℓ=5, the probability decreases to $3.07\times 10^{-3}$; and when ℓ=10, it further drops to $1.01\times 10^{-7}$. This implies that, for a practical system with N=64, a random guess by Eve is extremely unlikely to produce a chirp-parameter sequence close to the true one. In most cases, only a very small number of subcarriers are matched correctly, while the majority experience chirp-parameter mismatch, which severely disrupts the corresponding demodulation and detection processes.

#### 5.1.3. Dual-Layer Encryption Advantage

Combining the two mechanisms yields a total key space $K_{\mathrm{total}}=K_{\mathrm{idx}}\cdot K_{\mathrm{phase}}$, integrating factorial combinatorial growth with factorial parametric growth. The two layers protect distinct structural dimensions: IM conceals sparsity support in the affine-frequency domain, while chirp parameter encryption randomizes modulation-domain phase structure. These mechanisms are structurally independent; recovering one layer does not reveal the other. If the permutation is incorrect, index bits are randomized. If the quadratic phase parameters are incorrect, both index and modulation bits become statistically independent of the transmitted bits under optimal detection. Compared with index-only encryption, the dual-layer design eliminates residual modulation detectability; compared with phase-only encryption, it further enlarges the search space and strengthens resistance against brute-force attacks.

Consequently, the proposed dual-layer encrypted MIMO-AFDM-IM system achieves enhanced computational hardness and complete detection-level degradation under parameter mismatch while preserving the overall performance advantages of IM.

### 5.2. CRLB Analysis

To characterize the theoretical accuracy limit of the proposed sensing method, the Cramér–Rao lower bound (CRLB) is derived for the multi-parameter estimation problem. By stacking the vectorized sensing snapshots over $M_{R}$ AFDM sensing symbols, the observation vector is written as

(63) $$\psi ={\left[\mathrm{vec}{\left(Z_{1}\right)}^{T},\mathrm{vec}{\left(Z_{2}\right)}^{T},\ldots ,\mathrm{vec}{\left(Z_{M_{R}}\right)}^{T}\right]}^{T}=\bar{\psi }\left(\Phi \right)+w,$$

where $w∼\mathrm{CN}(0,\sigma^{2}I)$. The complete parameter vector is defined as

(64) Φ=ϑT,φT,τT,μT,Re{h}T,Im{h}TT.

Under the complex Gaussian observation model, the (i,j)-th entry of the Fisher information matrix (FIM) is

(65) [J(Φ)]i,j=2σ2Re∑m=1MR∂vec(Z¯m)∂ΦiH∂vec(Z¯m)∂Φj.

The required derivatives follow directly from the receive and transmit steering vectors, the delay-dependent phase term $D(\tau_{p})$, and the Doppler phase term $\exp(jmP_{S}\mu_{p})$. Specifically, the angle derivatives are obtained by differentiating the ULA steering vectors with respect to $\vartheta_{p}$ and $\varphi_{p}$, the delay derivative is obtained from the frequency-dependent phase rotation, and the Doppler derivative is obtained from the sensing-symbol phase progression over *m*. Since the complex path gains are nuisance parameters, define the parameter vector of interest as

(66) $$\rho ={\left[\vartheta^{T},\varphi^{T},\tau^{T},\mu^{T}\right]}^{T}.$$

Then the FIM can be partitioned as

(67) $$J\left(\Phi \right)=\left[\begin{array}{cc} J_{\rho \rho } & J_{\rho h}\\ J_{h\rho } & J_{hh} \end{array}\right],$$

and the equivalent FIM after eliminating the nuisance parameters is

(68) $$J_{\mathrm{eff}}=J_{\rho \rho }-J_{\rho h}J_{hh}^{-1}J_{h\rho }.$$

Accordingly, the CRLB is given by

(69) $$\mathrm{CRLB}\left(\rho \right)=J_{\mathrm{eff}}^{-1}.$$

Therefore, for the *p*-th path, the CRLBs of AoA, AoD, delay, and Doppler are respectively obtained from the diagonal entries of $J_{\mathrm{eff}}^{-1}$ as

(70) $$\begin{array}{cc} \; & \mathrm{CRLB}\left(\vartheta_{p}\right)={\left[J_{\mathrm{eff}}^{-1}\right]}_{p+1,p+1},\\ \; & \mathrm{CRLB}\left(\varphi_{p}\right)={\left[J_{\mathrm{eff}}^{-1}\right]}_{P+p+1,P+p+1},\\ \; & \mathrm{CRLB}\left(\tau_{p}\right)={\left[J_{\mathrm{eff}}^{-1}\right]}_{2P+p+1,2P+p+1},\\ \; & \mathrm{CRLB}\left(\mu_{p}\right)={\left[J_{\mathrm{eff}}^{-1}\right]}_{3P+p+1,3P+p+1}. \end{array}$$

The derived CRLB serves as the theoretical benchmark for the sensing RMSE curves presented in Section 6. The Doppler dependence of the CRLB can be interpreted from the FIM, where the Doppler parameter enters through the temporal phase progression $\exp(jmP_{S}\mu_{p})$. When the Doppler values lie within the unambiguous range and different paths have sufficient Doppler separation, the corresponding FIM remains well conditioned and the CRLB varies smoothly with Doppler. However, when Doppler values approach the ambiguity boundary or two paths have nearly identical Doppler signatures, the columns of the parameter Jacobian become more correlated, leading to a larger CRLB. This explains why velocity estimation is generally more sensitive than angular estimation in the low-SNR region.

### 5.3. BER Analysis

In this sub-section, we provide an ABEP analysis for a generalized non-encrypted MIMO-AFDM-IM extension under the spatial multiplexing framework, where each transmit antenna conveys an independent AFDM-IM block and the receiver jointly processes all spatial streams. This analysis is included to provide analytical insight into the pairwise error behavior of MIMO-AFDM-IM over effective delay–Doppler channels, rather than to serve as a closed-form BER proof for every operation in the complete encrypted ISAC transceiver. The practical system considered in the proposed secure ISAC receiver remains the single-stream beamformed architecture described in Section 4; it can be interpreted as an effective-channel specialization after beamforming and sensing-assisted reconstruction. The encryption layers are omitted in the analytical derivation because they are deterministic preprocessing operations for the legitimate receiver with the correct key, whereas wrong-key detection and sensing-error effects are evaluated numerically in the simulation section. For analytical tractability, the input–output relation can be rewritten as

(71) $$y_{r}=\sum_{t=1}^{N_{t}}\Phi \left(x_{t}\right)h^{\left[r,t\right]}+{\tilde{w}}_{r},$$

where $\Phi \left(x_{t}\right)\in C^{N\times P}$ is a concatenated matrix, given by $\Phi \left(x_{t}\right)=\left[H_{0}x_{t},\ldots ,H_{P-1}x_{t}\right]$, where $H_{p}≜\Lambda_{c_{2}}F\Lambda_{c_{1}}\Gamma_{{\mathrm{CPP}}_{p}}\Delta_{f_{p}}\Pi^{l_{p}}\Lambda_{c_{1}}^{H}F^{H}\Lambda_{c_{2}}^{H}$, for p=0,…,P−1.

Here, $H_{p}$ represents the channel matrix associated with the *p*-th path, and $h^{\left[r,t\right]}={\left[h_{0}^{\left[r,t\right]},\ldots ,h_{P-1}^{\left[r,t\right]}\right]}^{T}\in C^{P\times 1}$ is the fading coefficient vector between the *t*-th TA and *r*-th RA.

Considering all the RAs, the equivalent input–output relation of the scheme can be rewritten as

(72) $$y_{\mathrm{MIMO}}=\Theta \left(x_{\mathrm{MIMO}}\right)h_{\mathrm{MIMO}}+w_{\mathrm{eff}},$$

where

(73) $$\Theta \left(x_{\mathrm{MIMO}}\right)=I_{N_{r}}⊗\left[\Phi \left(x_{1}\right),\ldots ,\Phi \left(x_{N_{t}}\right)\right]\in C^{N_{r}N\times N_{r}N_{t}P},$$

(74) $$h_{\mathrm{MIMO}}={\left[{\left(h^{\left[1,1\right]}\right)}^{T},\ldots ,{\left(h^{\left[1,N_{t}\right]}\right)}^{T},\ldots ,{\left(h^{\left[N_{r},1\right]}\right)}^{T},\ldots ,{\left(h^{\left[N_{r},N_{t}\right]}\right)}^{T}\right]}^{T}\in C^{N_{r}N_{t}P\times 1}.$$

Assuming the availability of channel state information, the conditional pairwise error probability (CPEP) can be expressed as

(75) PxMIMO→x^MIMO∣hMIMO=PyMIMO−Θ(x^MIMO)hMIMO2<yMIMO−Θ(xMIMO)hMIMO2=P2ReweffHΔΘhMIMO<−ΔΘhMIMO2.

where $\Delta \Theta =\Theta \left({\hat{x}}_{\mathrm{MIMO}\;}\right)-\Theta \left(x_{\mathrm{MIMO}}\right)$ represents the matrix difference. Since 2ReweffHΔΘhMIMO follows a Gaussian distribution with variance $2N_{0}{∥\Delta \Theta h_{\mathrm{MIMO}}∥}^{2}$, the CPEP simplifies to

(76) $$P\;\left(x_{\mathrm{MIMO}}\to {\hat{x}}_{\mathrm{MIMO}}|h_{\mathrm{MIMO}}\right)=Q\;\left(\sqrt{\frac{{∥\Delta \Theta h_{\mathrm{MIMO}}∥}^{2}}{2N_{0}}}\right)=Q\;\left(\sqrt{\frac{h_{\mathrm{MIMO}}^{H}Ah_{\mathrm{MIMO}}}{2N_{0}}}\right).$$

where Q(x) denotes the Gaussian tail function and $A=\Delta \Theta^{H}\Delta \Theta$. Based on the approximate definition of the *Q* -function in Ref. [41], the CPEP can be approximated as

(77) $$P\left(x_{\mathrm{MIMO}}\to {\hat{x}}_{\mathrm{MIMO}}|h_{\mathrm{MIMO}}\right)≅\frac{1}{12}e^{-\delta /4N_{0}}+\frac{1}{4}e^{-\delta /3N_{0}}.$$

The above approximation converts the Gaussian tail probability into a weighted sum of exponential functions, which makes the subsequent channel averaging tractable. The unconditional PEP (UPEP) for the MIMO-AFDM-IM scheme is derived by averaging over the distribution of $\delta =h_{\mathrm{MIMO}\;}^{H}Ah_{\mathrm{MIMO}\;}$, which depends on the channel realization and matrix A. This results in the following expression for the UPEP

(78) $$P\left(x\to \hat{x}\right)≅E_{h}\left\{\frac{1}{12}e^{-\delta /4N_{0}}+\frac{1}{4}e^{-\delta /3N_{0}}\right\}.$$

By leveraging the properties of matrix A and applying results from the moment-generating function of quadratic forms, the expectation can be reformulated in terms of the eigenvalues of A. In particular, after eigenvalue decomposition of A, the quadratic form is expressed as a weighted sum over independent channel components, and the exponential expectation becomes a product of scalar moment-generating functions. Using eigenvalue decomposition, the UPEP becomes

(79) $$P\left(x\to \hat{x}\right)≅\frac{1}{12}\prod_{r=1}^{R}\frac{1}{1+\frac{\lambda_{r}^{\left(A\right)}}{4PN_{0}}}+\frac{1}{4}\prod_{r=1}^{R}\frac{1}{1+\frac{\lambda_{r}^{\left(A\right)}}{3PN_{0}}},$$

where *R* denotes the rank of A, and $\lambda_{r}^{\left(A\right)}$ are the eigenvalues of A.

The ABEP of the MIMO-AFDM-IM scheme can be approximated as

(80) $$P_{b}\approx \frac{1}{mn_{x}}\sum_{x}\sum_{x\neq \hat{x}}P\left(x\to \hat{x}\right)e\left(x,\hat{x}\right),$$

where $n_{x}$ is the total number of possible realizations of x, and $e(x,\hat{x})$ denotes the number of bit errors associated with each pairwise error event.

### 5.4. Spectral Efficiency and Computational Complexity

After analyzing the security and BER performance, we further evaluate the SE enabled by IM. Neglecting the overhead of the cyclic prefix for simplicity, the SE of the AFDM-IM scheme can be approximated as

(81) η≈log2nsubksub+ksublog2(M)nsub.

The first term corresponds to the information carried by subcarrier index selection, while the second term accounts for conventional constellation modulation. The above expression represents the payload SE of the AFDM-IM data symbols. If pilot, sensing, synchronization, and control overheads are included, an effective SE can be written as

(82) $$\eta_{\mathrm{eff}}=\left(1-\eta_{\mathrm{oh}}\right)\eta ,$$

where $\eta_{\mathrm{oh}}$ denotes the aggregate overhead ratio. Similarly, the EE improvement discussed in this paper is mainly associated with sparse subcarrier activation and communication reliability, while an implementation-level EE should account for transmit power, circuit power, and processing power, e.g.,

(83) $${\mathrm{EE}}_{\mathrm{eff}}=\frac{R_{\mathrm{eff}}}{P_{\mathrm{tx}}+P_{\mathrm{circuit}}+P_{\mathrm{proc}}}.$$

These overhead-aware metrics are useful for hardware-oriented evaluation, but the present analysis focuses on the waveform-level SE gain and the dominant computational complexity.

The overall computational complexity of the proposed ISAC framework consists of two major components: the cascaded multi-domain sensing procedure and the communication detection process. The sensing stage involves spatial, delay, and Doppler parameter estimation, whose dominant cost scales cubically with the receive antenna dimension due to covariance processing and subspace decomposition. The communication stage adopts MMSE-based equalization followed by sub-block-wise ML detection, resulting in polynomial complexity rather than exhaustive search over the full transmit vector.

The cascaded sensing algorithm can be further decomposed as follows. The AOA stage constructs the spatial covariance matrix and performs eigenvalue decomposition, with a dominant order of approximately $O(M_{R}NN_{r}^{2}+N_{r}^{3})$. The delay stage performs a one-dimensional search over a delay grid of size $N_{\tau }$ for each detected path, leading to approximately $O(P^{♯}N_{\tau }M_{R}NN_{r})$ operations. The AOD-Doppler stage relies on low-rank decomposition of the equivalent $N_{t}\times M_{R}$ matrix for each path, whose order is approximately $O(P^{♯}N_{t}M_{R}\min\left\{N_{t},M_{R}\right\})$. The final least-squares gain projection has a lower order of approximately $O(P^{♯}N_{t}M_{R})$. Therefore, the overall cascaded sensing complexity can be summarized as

(84) $$O\;\left(M_{R}NN_{r}^{2}+N_{r}^{3}+P^{♯}N_{\tau }M_{R}NN_{r}+P^{♯}N_{t}M_{R}\min\left\{N_{t},M_{R}\right\}\right).$$

This cascaded structure avoids direct high-dimensional joint search over AOA, delay, AOD, and Doppler grids. If the four parameter grids were searched jointly, the complexity would scale approximately as

(85) $$O\;\left(N_{\vartheta }N_{\tau }N_{\varphi }N_{\mu }M_{R}NN_{t}N_{r}\right).$$

The above expression also indicates the main sources of processing delay. The receive-array dimension $N_{r}$ affects both covariance accumulation and eigenvalue decomposition, with the latter scaling cubically with $N_{r}$. The delay-search latency grows linearly with the detected path number $P^{♯}$, delay-grid size $N_{\tau }$, subcarrier number *N*, and sensing-symbol number $M_{R}$. The AOD-Doppler reconstruction scales linearly with $P^{♯}$ and is governed by the smaller dimension of the $N_{t}\times M_{R}$ low-rank matrix. Therefore, increasing $N_{\tau }$ or $P^{♯}$ mainly increases the serial per-path delay, whereas increasing $N_{r}$ has a stronger impact on the subspace-processing latency. The covariance accumulation and per-path searches can be parallelized across subcarriers or paths, but the cascaded dependency among AOA, delay, and AOD-Doppler stages still introduces sequential processing delay.

For the communication receiver, MMSE equalization over an *N*-subcarrier AFDM block requires approximately $O(N^{3})$ operations per communication symbol if direct matrix inversion is used. The subsequent sub-block-wise ML detection has complexity O(GnsubksubMksub), where $G=N/n_{\mathrm{sub}}$ is the number of IM sub-blocks. Hence, over $M_{C}$ communication symbols, the dominant communication-side complexity is approximately

(86) OMCN3+GnsubksubMksub.

The total processing latency is consequently determined by the sequential sensing stages plus the communication equalization and detection stages. From an implementation viewpoint, the main latency bottlenecks are the receive-array eigendecomposition in MUSIC, the per-path delay search, the low-rank AOD-Doppler reconstruction, and the sub-block-wise ML detector. The covariance accumulation and per-path searches can be parallelized, and the ML detector may be replaced by list, sphere, or message-passing detection when lower latency is required. Therefore, the proposed receiver should be viewed as a performance-oriented benchmark for secure ISAC operation, while real-time hardware deployment requires further execution-time profiling and architecture-aware optimization.

## 6. Simulations Results

The simulations were conducted using MATLAB R2024b (MathWorks, Natick, MA, USA; https://www.mathworks.com/). This section evaluates the communication reliability and security performance of the proposed scheme based on Monte Carlo simulations. Unless otherwise specified, the following parameters are adopted. We consider a 200m×200m ISAC region, where the BS is located at the geometric center (origin of the coordinate system). The transceiver is equipped with $N_{t}=16$ transmit antennas and $N_{r}=16$ receive antennas. The carrier frequency is $f_{C}=60\;\mathrm{GHz}$ and the total system bandwidth is 100MHz. A total of N=64 subcarriers are employed with a subcarrier spacing of 1.5625MHz. The number of AFDM sensing symbols is $M_{R}=32$, and QPSK modulation is adopted. In the subcarrier index encryption scheme, each sub-block contains $n_{\mathrm{sub}}=4$ subcarriers, of which $k_{\mathrm{sub}}=2$ are activated for IM.

A doubly selective multipath channel with three propagation paths is considered, where the vehicle position, orientation angle, and reflector locations are randomly generated. The UE velocity vector is fixed as ${\left[6,\;9\right]}^{⊤}\;m/s$.

The pre-chirp parameter is configured as $c_{1}=\frac{2f_{\max}+1}{2N}$. In conventional AFDM systems, the quadratic coefficient $c_{2}$ is chosen either as an irrational number or as a rational number much smaller than $\frac{1}{2N}$. In contrast, the proposed scheme employs a specifically generated $c_{2}$ set for encryption purposes. Unless otherwise specified, the normalized fading baseline uses $\beta_{p}=1$, and the small-scale fading term $g_{p}$ is independently generated according to $g_{p}∼\mathrm{CN}\left(0,1/P\right)$ in each Monte Carlo channel realization. Both the legitimate receiver and the Eve experience channels generated according to the same statistical model. The legitimate receiver has access to the correct encryption parameters, whereas the Eve does not possess the undisclosed keys. Unless otherwise specified, the simulations adopt AWGN to isolate the waveform, sensing, and encryption effects from non-Gaussian disturbance factors. Impulsive noise and robust receiver design are not included in the present simulation setting.

**Figure 5:**
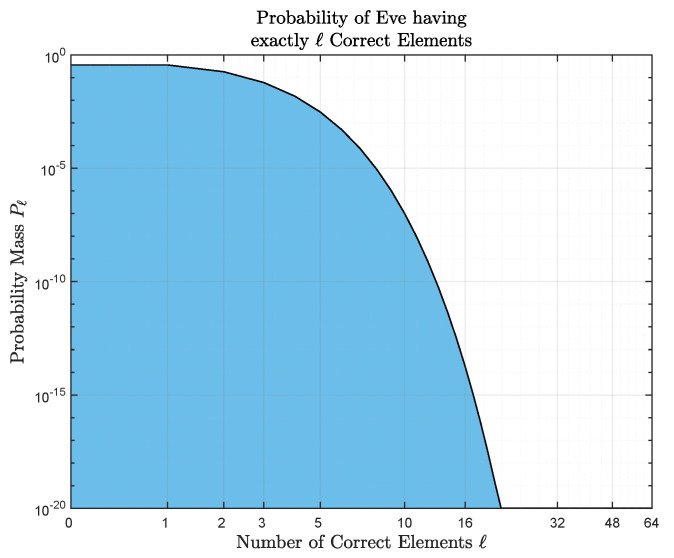
Probability distribution of the number of correctly guessed chirp-parameter positions at the eavesdropper.

To provide a more intuitive view of the security brought by chirp-parameter encryption, Figure 5 depicts the probability distribution that Eve randomly guesses a sequence containing exactly *ℓ* correctly matched chirp parameters. The plotted probabilities are obtained from (62) with N=64. It can be observed that the probability decreases rapidly as *ℓ* increases. For small *ℓ*, the probability is the highest but still remains below 0.37. When *ℓ* reaches a moderate value, e.g., ℓ≈5, the probability has already dropped to the order of $10^{-3}$. For larger *ℓ* values, such as ℓ≥10, the probability becomes nearly negligible. This trend indicates that, under random guessing, Eve is almost unable to obtain a chirp-parameter sequence close to the true permutation. As a result, most subcarriers suffer chirp-parameter mismatch, which introduces large-scale phase perturbations into the demodulation and detection process and thus significantly degrades the overall receiver performance. These probability values provide a numerical explanation for why blind recovery of the chirp-parameter permutation is infeasible when N=64.

Next, the impact of the second-layer encryption on the BER performance at the illegitimate receiver is investigated from two aspects: the upper bound of the quadratic coefficient $c_{2,\max}$ and the number of undisclosed parameters $K_{c_{2}}$ (corresponding to Section 2.4).

**Figure 6:**
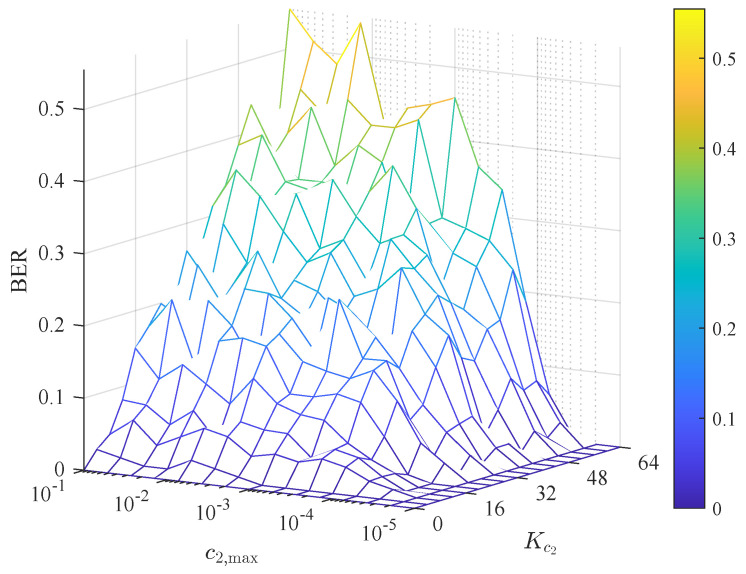
BER at the eavesdropper versus quadratic coefficient range $c_{2,\max}$ and number of undisclosed parameters $K_{c_{2}}$.

As shown in Figure 6, the parameter $c_{2,\max}$ is logarithmically selected within the range $\left[4.99\times 10^{-6},\;4.88\times 10^{-1}\right]$ with 10 sampling points, while $K_{c_{2}}$ increases from 0 to 64 with a step size of 4. Here, $K_{c_{2}}$ denotes the number of subcarrier-wise quadratic phase coefficients $c_{2}\left[m\right]$ that are undisclosed to the eavesdropper among the total *N* subcarriers. When $K_{c_{2}}$ is small, the Eve can still infer most of the modulation structure, resulting in a relatively low BER. As $K_{c_{2}}$ increases, the unknown quadratic phase rotations introduce severe structural distortion in the received signal, preventing the Eve from correctly reconstructing the AFDM modulation relationship. Consequently, the BER rapidly increases and approaches the random-guessing bound.

The parameter $c_{2,\max}$ determines the dynamic range of quadratic phase perturbations across subcarriers. For small $c_{2,\max}$, especially in the $10^{-5}$–$10^{-4}$ range, the induced phase variations are limited, leading to weak encryption gain. When $c_{2,\max}$ increases to approximately the order of $10^{-2}$, the quadratic phase rotations across subcarriers become sufficiently diverse, and the phase dispersion effect approaches saturation. Under this condition, increasing the number of undisclosed parameters toward $K_{c_{2}}=64$ drives the Eve’s BER close to the random-guessing level of 0.5. Further increasing $c_{2,\max}$ yields negligible additional security improvement, indicating that this magnitude provides an effective operating region for chirp-based encryption design.

**Figure 7:**
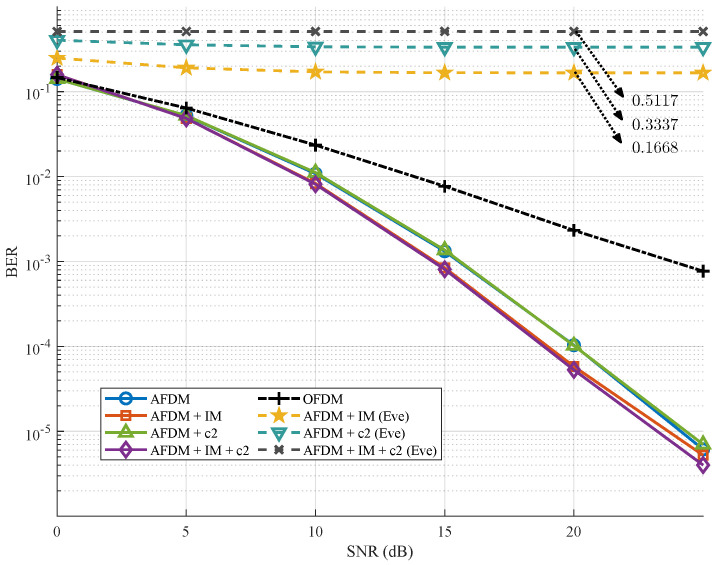
BER performance comparison of the proposed multi-layer encryption scheme and single-layer strategies at both legitimate and eavesdropper receivers.

Figure 7 compares the BER performance of the proposed multi-layer encryption with several single-layer strategies, alongside the corresponding BER at the Eve and benchmark AFDM and OFDM systems without encryption. The maximum chirp perturbation is set as $c_{2,\max}=4.6\times 10^{-3}$.

For the legitimate link, encrypted AFDM achieves nearly the same BER as non-encrypted AFDM, showing that encryption does not degrade reliability. Under doubly selective channels, AFDM outperforms OFDM due to its full-diversity property, maintaining robustness in mobile scenarios.

From the security perspective, single-layer encryption increases Eve’s BER: 0.3337 for chirp-parameter encryption and 0.1668 for subcarrier IM. Chirp encryption is more disruptive, as it directly modifies the signal structure rather than just masking subcarrier patterns. The proposed multi-layer scheme combining IM and chirp encryption raises Eve’s BER to 0.5117, significantly surpassing single-layer methods and confirming enhanced physical-layer security without affecting legitimate transmission.

Compared with the IM-only and chirp-only baselines, the dual-layer scheme increases the Eve’s BER by approximately 0.3449 and 0.1780, respectively.

**Figure 8:**
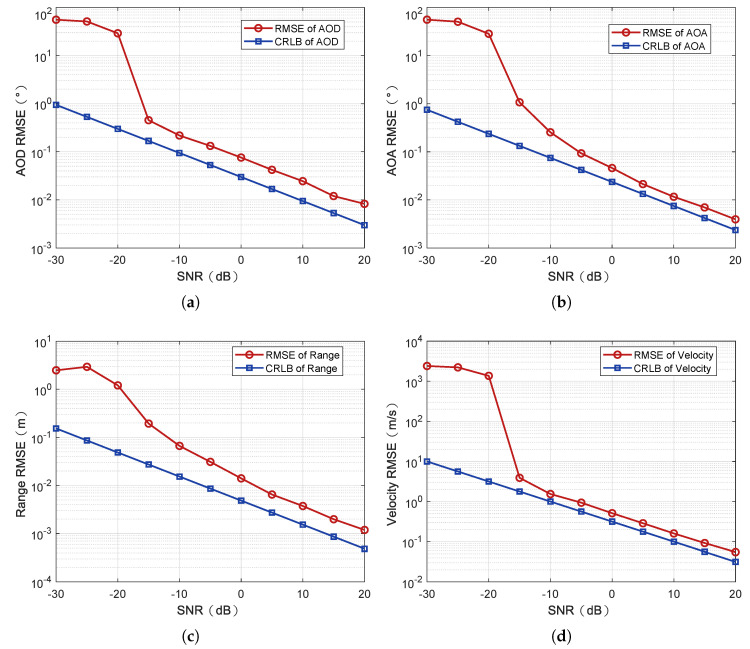
RMSE performance of the proposed sensing receiver: (**a**) AOD estimation; (**b**) AOA estimation; (**c**) range estimation; (**d**) velocity estimation.

The performances of the proposed sensing method and the corresponding CRLBs over different SNRs are shown in Figure 8. The RMSEs of AOD, AOA, range, and velocity decrease as SNR increases, approaching their respective CRLBs at high SNRs. In the low-SNR region, estimation errors are larger due to noise, while a noticeable improvement occurs when SNR exceeds 20 dB. This transition point is consistently observed in the four sensing metrics, indicating that the cascaded estimator becomes CRLB-consistent in the moderate-to-high SNR region. Among the parameters, range and velocity exhibit slightly larger deviations from the CRLB compared with angular parameters. Overall, the results demonstrate that the proposed method achieves near-optimal performance and is robust across the evaluated SNR range.

**Figure 9:**
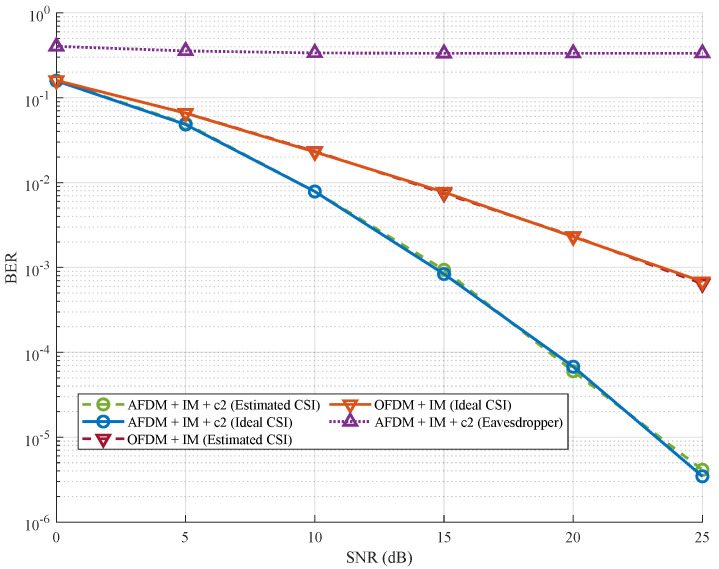
BER comparison of encrypted AFDM and OFDM waveforms under ideal and estimated channel conditions, including the corresponding eavesdropper performance.

Figure 9 presents the BER performance of the encrypted AFDM and OFDM systems under both ideal and estimated CSI conditions, together with the corresponding results at the Eve.

We first examine the impact of channel state information accuracy on the legitimate link. It can be observed that the BER curves under estimated CSI closely approach those obtained under ideal CSI for the encrypted AFDM system over the evaluated SNR range of 0–25 dB. This indicates that the designed sensing-assisted channel estimation scheme effectively supports the communication process. Even in the presence of CSI estimation errors, the system maintains near-ideal detection performance, demonstrating the robustness of the ISAC framework. This comparison also reflects the impact of sensing reconstruction error on the legitimate communication link, while detailed hardware-impairment modeling, such as phase noise or residual synchronization offsets, is outside the present simulation model.

Next, a comparison between AFDM and OFDM is conducted. Since both waveforms belong to multicarrier modulation frameworks, security enhancement can be introduced via subcarrier IM in both cases. However, unlike OFDM, which relies solely on index-based encryption, AFDM additionally offers controllable chirp parameters, enabling a dual-layer encryption mechanism based on both IM and chirp configuration. The results show that the proposed dual-layer encrypted AFDM scheme achieves superior BER performance compared with the OFDM scheme employing only index-based encryption. This performance advantage mainly stems from the capability of AFDM to achieve full diversity over doubly selective channels, thereby improving reliability in mobile and time-varying environments. The current comparison focuses on isolating the effects of AFDM, IM, chirp-parameter encryption, and their combination under a common simulation setting. A direct comparison with secure OTFS-based and OFDM-based ISAC designs and other physical-layer security methods requires matched pilot structures, channel estimators, receiver assumptions, and key-generation models.

From the Eve’s perspective, the absence of the correct index and chirp parameters prevents accurate signal reconstruction. As a result, the demodulation and detection processes are severely degraded, and the eavesdropper BER remains close to the random-decision level over the simulated SNR range. This confirms that the proposed dual-layer secure transmission scheme enhances physical-layer security while preserving reliable performance for the legitimate receiver.

## 7. Conclusions

In this paper, a multi-layer encryption MIMO-AFDM-IM scheme was proposed for secure mobile ISAC systems. By jointly exploiting the affine chirp parameter $c_{2}$ and subcarrier IM, a multi-dimensional physical-layer security mechanism was constructed without degrading delay–Doppler robustness. A unified downlink MIMO configuration was adopted to enable joint angle–delay–Doppler estimation, and the obtained sensing information was utilized to assist beamforming and signal equalization. Simulation results verify that the proposed design substantially degrades unauthorized detection while preserving reliable transmission for the legitimate receiver, and the sensing results remain consistent with the theoretical CRLB behavior in the high-SNR region. Future studies may further extend this framework by incorporating broader security metrics, practical implementation constraints, and more diverse deployment conditions.

## Figures and Tables

**Table 1:** Comparison of conventional and encrypted index mapping.

Bits $b_{\mathrm{idx}}$	Conventional $I_{t}^{g}$	Encrypted $I_{t}^{g}\left(K\right)$	AFDM-IM Sub-Block Structure
[00]	{1,2}	{1,4}	${\left[x_{t,1}^{g}\;x_{t,2}^{g}\;0\;0\right]}^{T}\to {\left[x_{t,1}^{g}\;0\;0\;x_{t,2}^{g}\right]}^{T}$
[01]	{1,3}	{1,2}	${\left[x_{t,1}^{g}\;0\;x_{t,2}^{g}\;0\right]}^{T}\to {\left[x_{t,1}^{g}\;x_{t,2}^{g}\;0\;0\right]}^{T}$
[10]	{1,4}	{2,3}	${\left[x_{t,1}^{g}\;0\;0\;x_{t,2}^{g}\right]}^{T}\to {\left[0\;x_{t,1}^{g}\;x_{t,2}^{g}\;0\right]}^{T}$
[11]	{2,3}	{1,3}	${\left[0\;x_{t,1}^{g}\;x_{t,2}^{g}\;0\right]}^{T}\to {\left[x_{t,1}^{g}\;0\;x_{t,2}^{g}\;0\right]}^{T}$

## Data Availability

Data are contained within the article.
